# Diversity, equity, inclusion, and access are necessary for clinical trial site readiness

**DOI:** 10.1017/cts.2023.660

**Published:** 2023-11-30

**Authors:** Lori Carter-Edwards, Bertha Hidalgo, Freda Lewis-Hall, Tung Nguyen, Joni Rutter

**Affiliations:** 1 Kaiser Permanente Bernard J Tyson School of Medicine, Pasadena, CA, USA; 2 The University of North Carolina at Chapel Hill Gillings School of Global Public Health, Chapel Hill, NC, USA; 3 The University of Alabama at Birmingham, Birmingham, AL, USA; 4 Retired from Pfizer Inc., New York, USA; 5 University of California San Francisco, San Francisco, CA, USA; 6 National Center for Advancing Translational Sciences, Bethesda, MD, USA

**Keywords:** Clinical trial sites, site readiness practices, quality improvement, evaluation, adoption and implementation, diversity, equity, inclusion, and access

Clinical research has long struggled with diversity, equity, inclusion, and access (DEIA). Despite the increasing diversity of the U.S. population, marginalized groups continue to be underrepresented in clinical trials. The lack of representation in clinical trials has impeded innovation, compromised generalizability of evidence, and may undermine trust in the clinical trials enterprise [1] – conclusions that FDA echoes in its guidance on diversity plans [2]. DEIA must inform clinical trials from design to dissemination to generate valid, generalizable evidence that meets the needs of an increasingly diverse population and addresses health inequities.

In this commentary, we discuss select site readiness practices, outlined in a companion article [3], which are particularly relevant to promoting DEIA in clinical trials. This perspective focuses on race and ethnicity, though other aspects of diversity, such as ability, sex, gender, sexual orientation, and others, also are underrepresented in clinical research [1,4]. Addressing race and ethnicity is an opportunity to intentionally focus efforts on a long-standing area of exclusion in clinical trials. The companion article includes the full set of site readiness practices, methods for their selection, and a rationale for their use by clinical trial sites. Site readiness practices must include DEIA considerations to avoid perpetuating existing inequities and to help address known or suspected barriers to clinical trials access – such as language, transportation, or dependent care [5]. Additionally, site readiness practices should seek to address structural constructs perpetuated within the medical and clinical trial enterprise that impede progress on these issues – through insurance coverage, hiring practices, partnership with communities, and mentorship opportunities. To this point, improving DEIA should specifically and explicitly be expressed as both goals and metrics for progress. Effective DEIA efforts in research participation should begin early, during the design and budgeting stages. Site readiness often involves actions taken even before the project’s conceptualization, such as modifying existing systems or policies. We believe that the site readiness practices discussed in this article (Table 1) support the development of a clinical research system that is intentionally inclusive, and therefore more closely aligned with the needs of patients.

**Table 1.** Site readiness practices that promote clinical trial site readiness through diversity, equity, inclusion, and access

Site readiness practice	Reference domain
*Diversifying participants*	Research Team – Site Readiness Practice 1
The research team has sufficient and diverse personnel, to support the roles and functions needed to conduct a clinical trial and enroll trial participants who accurately reflect the patient population for the disease or condition being studied, with particular consideration for underrepresented and underserved groups.	
*Diversifying and sustaining research teams and workforce*	Infrastructure – Site Readiness Practice 11
Ensure sufficient processes for hiring and supporting diverse staff to fulfill the roles and functions needed to conduct a clinical trial (e.g., principal/subinvestigators, clinical research associates, research nurses, data managers, and study coordinators).	
*Emphasizing accountability in trial recruitment and retention*	Study Management – Site Readiness Practice 4
Research team has access to and process for recruiting and retaining eligible study participants, which should include a plan for enrolling adequate numbers of participants from populations that are underrepresented and underserved in clinical trials.	
*Protecting trial participants*	Ethics and Safety – Site Readiness Practice 1
Research team can protect the rights and welfare of trial participants pursuant to national, state, local, and other applicable requirements and study protocol.	
*Reducing bias through data privacy and transparency*	Data Collection and Management – Site Readiness Practice 5
Research team can ensure blinding/masking, while promoting transparency and trust with study participants regarding how their data will be used and who will have access to it.	

## Diversifying Participants

Ensure that research teams can enroll diverse trial participants by engaging communities early and incorporating community members to serve as advisors [6]. For example, to recruit diverse participants, a research team should include individuals who are proficient in languages commonly spoken by populations of interest and/or represent the community experience via race, ethnicity, or culture. Research team diversity can improve trust, particularly for populations that may justifiably associate white authority figures with oppression based on historical and current events. Further, community members or advisory boards can help the research teams ensure that the trials conducted will have an authentic, viable connection to the communities being asked to participate [7]. For example, they can assist in providing a clear understanding of the purpose and goals of the clinical trials in a respectful and culturally appropriate manner and develop effective solutions not based on stereotypes when unanticipated challenges arise. Ultimately, institutional infrastructure and leadership that prioritizes DEIA are crucial for ensuring the diversity of research teams and trial participants.

## Diversifying and Sustaining Research Teams and Workforce

Improve diversity and retention in the clinical trial workforce. This effort is a strong emphasis for the Clinical and Translational Science Award program [8]. While acknowledging the challenges of recruiting a representative workforce, it is essential to prioritize diverse participant recruitment for more impactful and generalizable scientific outcomes [8]. Institutions that have difficulty recruiting and retaining diverse workers will likely have trouble recruiting diverse participants. The ability to work with diverse participants should be evaluated as a core job responsibility during hiring and employment. It is more efficient to train diverse candidates in research methods than to train nondiverse candidates in language and cultural skills. A diverse research team is not just a strength but a necessity. Members should be fully engaged and empowered, not merely serving as tokens to demonstrate a commitment to diversity. Partnering with and hiring from minority-serving institutions, and diverse community organizations, and their networks can help expand the pool of candidates and build capacity to train diverse personnel [9]. Building community capacity through strategic partnerships can prepare the future clinical trials workforce, promote bidirectional knowledge sharing, and ensure the sustainability of DEIA efforts [10].

## Emphasizing Engagement in Trial Recruitment and Retention

Research studies lack external validity if the research participants do not reflect the diversity of the intended patient population. Medical journals are beginning to require authors to include demographics of the study population in publications [11]. Likewise, studies should have clear recruitment goals for diverse participants and a detailed plan on how to achieve them – a requirement that became law in the most recent appropriations act [12]. Economic inequalities in the United States are highly linked to race and ethnicity; economic barriers to trial participation, such as costs of transportation, living and lost wages, and caregiving, and ease of reimbursement mechanisms, should be considered when budgeting for a study that seeks to enroll diverse participants. Furthermore, researchers who recruit clinical trial participants from underrepresented communities but never return to share study outcomes with the community may drive members of these communities from participating in future clinical trials [13]. Efforts to improve recruitment and retention [14] and follow-up [15] for underrepresented communities, including individuals with inconsistent access to the healthcare system, must be included in clinical trial planning.

## Protecting Trial Participants

Employ privacy and security protections for research participants, accounting for the needs of diverse populations, including respect for tribal autonomy. Consent documents and discussions should be clear, culturally appropriate, and at an appropriate reading level [16]. Developing informed consent language in partnership with community members can help promote mutual understanding and trust between trial participants, researchers, and institutions [17].

## Promoting Data Transparency and Unbiased Data Collection and Analysis Practices

Data sharing policies that do not examine risks that minorities face from racial profiling, or consider historical injustices and health inequities [18] in how data are collected and shared [19] may reduce diversity in participation. Unbiased data collection, analysis, transparency, and dissemination are key to both scientific rigor and community trust, especially for building confidence in scientific findings. Data collection practices that use standards and inclusively represent minoritized populations are critical for ensuring data can be generalizable to the populations the disease affects. Good data capture and algorithm practices can reduce bias that may otherwise be introduced in the collection and analysis of data and allow more informed research conclusions that can deliver better health outcomes. Predefined data plans should tackle key issues like the optimal data collection method for the research question and potential statistical biases, such as the implications of using a p-value of 0.05. Given that data interpretation is susceptible to researcher bias, it is vital to involve a diverse group of data interpreters.

## What Can You Do?

These site readiness practices should serve as a basic DEIA roadmap and reference for every study and study site. However, we recognize that many study sites that are needed for diversity in research participation – such as non-R1 research institutions (as defined by the Carnegie Classification of Institutions of Higher Education), community-based sites, or rural sites – may not have the resources or capacity to meet all site readiness practices (Table 2). These are structural issues that need continual assessment, but there may be useful resources already within reach. For example, there may be local organizations that could help access diverse communities. In addition, there may be opportunities to partner with academic institutions, private companies, or other organizations to expand engagement with potential trial participants. Clinical and Translational Science Award (CTSA) hubs [20] could provide support through their integrating special population cores. Finally, recruitment methods that are cost effective, such as social media platforms, can also be effective tools for reaching diverse communities.

**Table 2.** List of site readiness practices for clinical trials, organized by domain

Domain	Site readiness practices
Research team	1. The research team has sufficient and diverse personnel, to support the roles and functions needed to conduct a clinical trial and enroll trial participants who accurately reflect the patient population for the disease or condition being studied, with particular consideration for underrepresented and underserved groups.
	2. The Principal Investigator is qualified through experience, training, and mentorship to lead and conduct clinical trials, and is free from regulatory debarment and other disciplinary actions that would prevent them from practicing medicine and conducting clinical research.
	3. Subinvestigators and other research team members are qualified through experience, training, and mentorship to conduct clinical trials are well trained in cultural humility and strategies for engaging with underrepresented communities, and free from disciplinary actions that would prevent them from conducting clinical trials.
	4. All research team members receive initial and refresher training to perform clinical trial activities per ICH GCP standards, and as appropriate, have received training that is tailored to an individual’s role and specific to the study protocol.
Infrastructure	1. Identify all satellite sites, external and community facilities, and contractors utilized to fulfill the requirements of studies.
	2. Ensure facilities (including satellite sites, external facilities, and contractors) and equipment are adequate to fulfill the requirements of a study.
	3. Provide reliable physical and operational infrastructure (e.g., electric power, internet access, telephone, email, and communications).
	4. Along with community affiliates, store documents, materials, product, and equipment in a secure location protected against theft, damage, tampering, or other harms during the duration of a study.
	5. Retain study records after the conclusion of a study pursuant to national, state, local, and other applicable requirements and study protocol.
	6. Safeguard staff and participants and secure virtual and physical assets (e.g., facilities, records, specimens) during a disruption of operations (e.g., natural disaster).
	7. Maintain essential functions after a major disruption of operations (e.g., natural disaster).
	8. Protect computers, networks, programs, and data from digital disruptions and attacks.
	9. Maintain interoperable information systems and technology capabilities (e.g. data standards, quality control), adequate to support clinical trial conduct.
	10. Initiate study (e.g., execute a contract) in a prompt manner.
	11. Ensure sufficient processes for hiring and supporting diverse staff to fulfill the roles and functions needed to conduct a clinical trial (e.g., principal/subinvestigators, clinical research associates, research nurses, data managers, and study coordinators).
	12. Identify and manage conflicts of interest, including complete financial disclosures for research team members, pursuant to national, state, local, and other applicable requirements and study protocol.
Study management	1. Research team utilizes standard operating procedures/processes for the conduct of clinical trials pursuant to national, state, local, and other applicable requirements and study protocol.
	2. Principal investigator monitors and can demonstrate oversight for all study-related activities, including those functions delegated to satellite sites and contractors, including recruitment, enrollment, and retention suitable for reflecting the diversity of the populations affected by the disease or intervention of study.
	3. Research team can execute study initiation, start-up, and close-out procedures in a prompt manner.
	4. Research team has access to and process for recruiting and retaining eligible study participants, which should include a plan for enrolling adequate numbers of participants from populations that are underrepresented and underserved in clinical trials.
	5. Research team can collect, handle, label, store, and ship digital and biological samples (e.g., cultures, blood, serum, plasma, urine, feces, tissues, and imaging) with appropriate documentation pursuant to national, state, local, and other applicable requirements and study protocol.
	6. Research team can handle investigational medical products, devices, and other means of intervention safely and securely and can record receipt, expiry, reconstitution, handling, dispensation, transfer, and/or destruction.
	7. Research team can establish, maintain, and record calibration for study specific equipment.
	8. Research team can maintain essential study documentation before, during, and after a trial.
	9. Responsible party^a^ must report study results to clinicaltrials.gov within the required times before, during, and after the conclusion of a study and has a strategy for dissemination of research findings to stakeholders and participants.
Data collection and management	1. Research team implements controls (e.g., audits, system validations, audit trails, electronic signatures, and documentation) for software and systems involved in processing study-related data pursuant to national, state, local, and other applicable requirements and study protocol.
	2. Research team can collect, access, retrieve, and exchange data in a timely, accurate, and complete manner.
	3. Monitors, sponsor personnel, and regulatory authorities have access to source material, electronic data systems, facilities, and source documents.
	4. Research team can ensure quality control of data and source documentation to ensure the integrity and proper reporting of study data.
	5. Research team can ensure blinding/masking, while promoting transparency and trust with study participants regarding how their data will be used and who will have access to it.
Quality oversight	1. Research team can ensure and verify that the quality requirements have been fulfilled pursuant to national, state, local, and other applicable requirements and study protocol.
	2. Research team members are able and empowered to identify, prevent, report, and correct safety and quality issues in a timely fashion.
	3. Research team can identify and remediate deficiency findings from regulatory inspections and sponsor audits (e.g., warning letters, FDA Form 483, corrective and preventive action).
Ethics and safety	1. Research team can protect the rights and welfare of trial participants pursuant to national, state, local, and other applicable requirements and study protocol.
	2. Research team can identify, assess, process, and report safety events (e.g., deviations, malfunctions, deficiencies, adverse events) pursuant to national, state, local, and other applicable requirements and study protocol.
	3. Research team can execute an informed consent/assent process that is respectful of participants and pursuant to national, state, local, and other applicable requirements and study protocol.
	4. Research team can maintain confidentiality for study participants, while promoting transparency and trust with participants regarding how their data will be used and who will have access to it.
	5. Research team has access to and reports to a properly constituted IRB/ethics committee pursuant to national, state, local, and other applicable requirements and study protocol.
	6. Research team engages with study participants, especially vulnerable populations (e.g., children, refugees, people with an intellectual or developmental disability) and populations that have experienced medical abuse and exploitation (e.g., racial and ethnic minorities), in an ethical and culturally appropriate manner, and addresses institutional racism through intentional recruitment and engagement strategies.
	7. Research team clearly communicates study risks and benefits to study participants in a manner that is accessible and culturally/linguistically appropriate.

a For more information on who qualifies as a responsible party, see https://www.ecfr.gov/current/title-42/chapter-I/subchapter-A/part-11 (accessed August 15, 2022).

Taking steps to implement these site readiness practices or using them to improve upon existing ones is a good first step, but only a starting point. Transformation of the field is needed to significantly advance representative clinical trials. Structural barriers remain for which other solutions will be needed. It is important to consider building an ecosystem that incorporates stakeholder, specifically communities, accountability for these site readiness practices to be most effective. Implementing the site readiness practices requires engagement from all levels of leadership, with clear goals and metrics to measure progress and success, and to identify areas in need of improvement. The DEIA Learning System Framework provides institutions with a guide for developing and implementing infrastructure for supporting the types of DEIA actions that the site readiness practices promote, and operationalizing definitions for diversity, equity, inclusion, and access [21]. Through deliberate progress toward these site readiness practices, clinical trials will be better designed to recognize and address health inequities.
